# Microarray gene expression profiling of neural tissues in bovine spastic paresis

**DOI:** 10.1186/1746-6148-9-122

**Published:** 2013-06-19

**Authors:** Lorraine Pariset, Silvia Bongiorni, Susana Bueno, Cesare EM Gruber, Gianluca Prosperini, Giovanni Chillemi, Silvia Bicorgna, Arcangelo Gentile, Alessio Valentini

**Affiliations:** 1 Department for Innovation in Biological, Agro-food and Forest systems (DIBAF), University of Tuscia, via S. Camillo de Lellis s.n.c, Viterbo, 01100, Italy; 2 CASPUR, (Inter-University Consortium for the Application of Super-Computing for Universities and Research), via dei Tizii 6, Rome 00185, Italy; 3 Department of Veterinary Medical Sciences, University of Bologna, via Tolara di Sopra 50, 40064, Bologna, Ozzano Emilia, Italy

## Abstract

**Background:**

Bovine Spastic Paresis (BSP) is a neuromuscular disorder which affects both male and female cattle. BSP is characterized by spastic contraction and overextension of the gastrocnemious muscle of one or both limbs and is associated with a scarce increase in body weight. This disease seems to be caused by an autosomal and recessive gene, with incomplete penetration, although no genes clearly involved with its onset have been so far identified. We employed cDNA microarrays to identify metabolic pathways affected by BSP in Romagnola cattle breed. Investigation of those pathways at the genome level can help to understand this disease.

**Results:**

Microarray analysis of control and affected individuals resulted in 268 differentially expressed genes. These genes were subjected to KEGG pathway functional clustering analysis, revealing that they are predominantly involved in Cell Communication, Signalling Molecules and Interaction and Signal Transduction, Diseases and Nervous System classes. Significantly enriched KEGG pathway’s classes for the differentially expressed genes were calculated; interestingly, all those significantly under-expressed in the affected samples are included in Neurodegenerative Diseases. To identify genome locations possibly harbouring gene(s) involved in the disease, the chromosome distribution of the differentially expressed genes was also investigated.

**Conclusions:**

The cDNA microarray we used in this study contains a brain library and, even if carrying an incomplete transcriptome representation, it has proven to be a valuable tool allowing us to add useful and new information to a poorly studied disease. By using this tool, we examined nearly 15000 transcripts and analysed gene pathways affected by the disease. Particularly, our data suggest also a defective glycinergic synaptic transmission in the development of the disease and an alteration of calcium signalling proteins. We provide data to acquire knowledge of a genetic disease for which literature still presents poor results and that could be further and specifically analysed in the next future. Moreover this study, performed in livestock, may also harbour molecular information useful for understanding human diseases.

## Background

The Bovine Spastic Paresis (BSP) is a neuromuscular disorder known since the twenties of past century, affecting both males and females. BSP is characterized by an overextension of the gastrocnemious muscle and linked to a scarce increase in body weight [1]. Although the genes involved have not been identified so far, the current hypothesis is that the disease is due a single autosomal recessive mutation with incomplete penetrance [2]. The BSP symptoms are similar to those of human hyperekplexia (OMIM ID #149400), a disease caused by mutations in genes encoding glycinergic proteins (*GLRA1*, OMIM ID #138491; *SLC6A5*, OMIM ID # 604159; *GLRB*, OMIM ID #138492; *GPHN*, OMIM ID #603930; *ARHGEF9*, OMIM ID #300429). Besides the economic importance in agriculture, BSP may therefore constitute a model also for the human disease.

We studied BSP in Romagnola cattle breed, which presents the disease with a frequency of 0.6% [2]. Since BSP is reported to be associated to the nervous system [3,4], we performed a microarray analysis on cDNA obtained from spinal cord of normal and affected cattle, in order to identify metabolic pathways associated with the disease. The aim of this study was to detect variations in gene pathways by correlating gene expression profiles of healthy and affected animals and to spot genome regions where genes responsible for the disease are more likely to map. This can help in pinpointing a number of genes potentially involved in the disease, providing suitable data for a future gene scan approach.

## Methods

### Samples

Samples of spinal cord of affected and control individuals of Romagnola breed, males and females, were collected from C6 to L6 immediately after slaughtering and later stored in RNA (Sigma-Aldrich). We expected to find most of the genes involved in the spinal cord, being the disease linked to the nervous system [3,4]. The experimental work was approved by the Animal Ethics Committee of CRA, Italy, in accordance with local ethical requirements. Total RNA was extracted from 250 μg tissue samples by homogenization in QIAzolLysis Reagent (Qiagen) and according to RNeasy Lipid kit (Qiagen) protocol. After purification, RNA integrity was determined spectroscopically (GeneQuantpro, Amersham Pharmacia Biotech) and by gel electrophoresis. Only RNA with A260/280 and A260/230 ratios above 1.8 was used for amplification. RNA was quantified using a DTX 800 Multimode Detector (Beckman Coulter) using quant-it kit (Invitrogen). For microarray analysis, RNA aliquots of 4 controls and 4 affected samples were pooled to reduce the template needed and to exclude differences in expression due to individual variation and not related to BSP [5]. For qRT-PCR, individual RNA samples from the same animal and tissue samples were quantified as described above and used for the amplification.

### Microarray experiments

Microarray slides, containing about 15 k cDNA replicated twice, were obtained by ARK genomics (The Roslin Institute & R(D)SVS, University of Edinburgh, Scotland). They were chosen for containing spots from a bovine brain library [6], a bovine monocyte library [7] and 979 control spots (landing lights, blank, salmon sperm, housekeeping genes). As stated in the previous section, the disease is likely to be linked to the nervous system [3,4]. Within each slide all spots are printed in duplicate; the experiment included two technical replicates; in each replicate two slides were duplicated in a dye-swap experiment [8]. We subsequently obtained a total of 8 replicates per cDNA spot. Inverse transcription was realized by SuperScript Indirect cDNA Labelling System (Invitrogen). Fluorescently-labeledcRNA was prepared using the 3DNA Array 900 MPX Cy3/Cy5 Kit (Genisphere), according to the manufacturer’s protocol. Pooled cDNA probes of 4 control and 4 affected animals were hybridised onto each bovine cDNA slide, according to Genisphere’s instructions. In short, 2 μg of total RNA was reverse transcribed using RT dT primer. Then, the cDNA and the fluorescent 3DNA reagent were hybridized to the microarray in succession. The fluorescent 3DNA reagent (Cy3 and Cy5) hybridize to the cDNA by a “capture sequence” complementary to a sequence on the 5’ end of the RT primer. Hybridization was performed at 60°C for 16 hours in a hybridization oven with shaking. Upon hybridization, microarrays were washed and dried according to the Genisphere’s instructions and fluorescence measurements were performed using a ScanArrayLite (Perkin Elmer) laser scanner.

### Quantitative real time PCR

RNA from the same animal and tissue samples were used to validate the microarray data. 1 μg of total RNA from affected and control samples was reverse transcribed into cDNA using a blend of oligo-dT and random hexanucleotides and the QuantiscriptReverse Transcriptase (Quiagen). cDNA was transcribed in pools, using two control and two affected samples. Before making the qRT-PCR, a PCR was performed on all cDNA samples to assess the quality of the cDNA. A standard curve was generated using serial dilutions of the cDNA to calculate the efficiency of amplification. Real-time quantitative PCR was set up for three genes (*ATP6V01*, *S100A12* and *BCL2L1*). Primer pairs, designed using primer3 [9], are listed in Additional file 1: Table S1 and are derived from publically available bovine ESTs. qRT-PCR was performed with the Brilliant II SYBR Green qRT-PCR kit (Stratagene, Agilent technologies) and using a Stratagene Mx3005P (Stratagene). Amplification results of affected samples were calibrated against those of controls to obtain the differences in gene expression. All results were normalized to ß-actin. Three biological replicates and two technical replicates were performed for each gene investigated.

### Polymerase chain reaction (PCR) conditions and sequence analysis

PCR primers for *ATP2A1/SERCA* and *SLC6A5* (Additional file 1: Table S1) were designed using primer3 [9] from the sequences available in Genbank (*ATP2A1* NC_007326.4; *SLC6A5* NC_007330.4) to amplify genomic fragments including mutations in the exons 6 and 16 for *ATP2A1* and in exons 3 and 4 for *SLC6A5*. Each polymerase chain reaction (PCR) was performed in a total volume of 20 μl containing 20 ng of genomic DNA, 1.0 pMol of each primer, 10 μl of BioMix (Bioline) composed by BIOTAQDNA Polymerase and 2 mM of dNTPs, on a PTC-100TM Peltier Thermal Cycler (MJ Research). A 5 minutes denaturation step was followed by 34 cycles of denaturation at 94°C (30 sec), annealing at 59°C (30 sec) and extension at 72°C (1 min); the final extension step was carried out at 72°C for 5 minutes. PCR products were purified through ExoSap-IT (USB Corporation) to remove residual primers and dNTPs and used as templates for forward and reverse sequencing reactions. Sequencing was performed using a ceq8800 sequencer using DTCS QuickStart Kit and purifying with AgencourtCleanSEQ 96 (Beckman Coulter), according to manufacturer instructions. Sequences were aligned with Bioedit software [10].

### Data analysis

Images were obtained by a ScanArrayLite (Perkin Elmer) laser scanner using Spotfinder software (TIGR). cDNA spots were automatically segmented, total foreground and background intensities of the two dyes were calculated for each spot. The spots were flagged by the software when they exhibited poor hybridization signals or when they were saturated. Spots with signal to background ratio below 1.5 were filtered out, together with flagged spots. Data systematic bias was removed by applying the dye-swap normalization [8,11] that uses the reverse labelling in the microarray replicates and the lowness normalization [12,13]. The subtraction of the local spot background signal from the foreground signal was done according to the method developed by Scharpf and coauthors [14]. To establish the significance of the differential expression for each probe the t-test, was used, correcting p-values for multiple tests [15]. Finally, only genes with absolute value of the fold change at least 2.0 and a p-value < 0.05 were considered.

KEGG pathways were retrieved for all slide genes [16-18].

Real Time data were analysed using the accompanying MxPro software (Stratagene). Relative expression of each gene for each sample was calculated by comparing Ct values of the target gene with Ct values of the ß-actin constitutive gene product. T-test was performed in order to assess the statistical significance of the RT-PCR results.

### Functional analysis

Functional analysis of gene lists was performed using the set of web-based functional annotation tools DAVID v6.7 [19,20]. The clustering tool was first used to check slide genes coverage and then to compare DEG pathways with the genes spotted on the slide. Furthermore hypergeometric test was computed to compare distribution in C1 class of KEGG pathways with genes spotted on slide. Then, DAVID tool was used to look for functional enrichment for genes over- and under-expressed more than two-fold in our samples. The statistical significance of the enrichment for the KEGG pathways of interest was computed by DAVID using the Fisher Exact test. KEGG pathway was selected as the functional annotation category for these analysis, assuming minimum number of genes for the corresponding C3 term (Count Threshold) = 2 and maximum EASE Score (modified Fisher P-value Threshold) = 0.005 for enrichment analysis, and CT = 1 and PT = 1 for DEGs versus total genes on slide.

## Results

### Differentially expressed genes

To identify genes possibly involved in BSP development, we performed an experiment using a bovine cDNA microarray by hybridising cDNA of affected and control Romagnola samples. Differentially Expressed Genes (DEGs) were identified using t-test statistics. A p-value <0.05, corrected for multiple tests, and an absolute Fold Change value (|FC|) ≥2.0 were used as criteria for the identification of DEGs. After filtering, 268 genes were found significantly over/under expressed in affected animals versus healthy controls; DEGs were annotated on Btau_4.0 version of the bovine genome, see Additional file 2: Table S2. The search in genomic databases was repeated several times during manuscript preparation to include sequences recently added in NCBI. The data have been deposited in NCBI Gene Expression Omnibus (GEO, http://www.ncbi.nlm.nih.gov/geo/info/linking.html) and are accessible through GEO, series accession number GSE25243. To validate microarray results, the expression of three genes (*ATP6V01*, *S100A12* and *BCL2L1*), significantly over-expressed in affected samples, were tested by Quantitative Real Time PCR (qRT-PCR). The qRT-PCR data confirmed that the genes were significantly over-expressed in affected with respect to control samples (Table 1).

**Table 1:** Comparison of microarray and qRT-PCR results

**Gene**	**Microarray**	**qRT-PCR**		
	***FC***	***P value***	***FC***	***P value***
*BCL2L1*	2.34	0.0377	10.24	0.00017
*S100A12*	23.71	0.0055	15.76	0.00022
*ATP6V01*	81.29	0.0008	6.30	0.01775

In the affected samples we observed a over-expression of the following genes: *ATP6V0E1* which is involved in Oxidative Phosphorylatin pathway (FC 81.25); *CATHL1* (FC 21.65) which is linked to Organismal Systems and Infectious Diseases pathway; the calcium binding protein *S100A12* (FC 23.71) which is involved in the Calcium Signalling pathway; the *HIST2H2AC* (FC 5.90) which is involved in the Immune System Disease class; and the ATP/GTP binding protein-like gene *AGBL5* (FC 3.67). Under-expression in affected samples was less marked (minimum value of FC -3.3). Among the under-expressed genes we observed the chromatin regulator *SMARCA5* (FC -2.92); the transcription factor *CREBZF* (FC -2.83). The Rho GDP dissociation inhibitor (GDI) alpha (*ARHGDIA*) is under-expressed in affected samples (FC -2.185). Two neurotransmitter transporters (*SLC41A3*, FC 2.361; *SLC25A20*, FC -2.191) and one organic anion transporter of the solute carrier family (*SLCO1A2*, FC -2.414) resulted altered in affected animals. Moreover, in affected samples we observed the over-expression of *GRINA* (FC 1.97) and *SLC6A9* (FC 1.9), two genes involved in glycinergic synaptic transmission. The *GFAP* gene, encoding for the glial fibrillary acidic protein, was also over-expressed in affected animals (FC 2.60). The *NR4A2* (FC 3.06) gene, a nuclear receptor family protein associated with dopaminergic dysfunctions, was over-expressed in affected animals.

We investigated the distribution of the DEGs in the bovine genome (UMD3.1 version). All *Bos taurus* chromosomes (BTAs) harbour differentially expressed genes; and a 30% percentage of them, is found in chromosomes 2, 3, 5, 7 and 18. We found over- and under-expressed gene clusters in narrow regions on BTA3, BTA5, BTA18 BTA21 and BTA22. Anyway, we have computed the χ2 statistics and we have not found any significant clustering of DEGs in these specific regions.

### Gene expression and KEGG analysis

The Kyoto Encyclopedia of Genes and Genomes (KEGG) database was used to link microarray results to KEGG pathways to their relative C1, C2 and C3 classes (Table 2). Of the 268 DEGs in the microarray experiment, 195 were annotated sequences, linked to KEGG pathways and to their relative classes; 29 represented unidentified transcripts and 22 encoded for proteins with poorly known or unknown function. The NCBI web-based functional annotation tool DAVID v. 6.7 (Database for Annotation, Visualization and Integrated Discovery) was used to investigate functional associations of gene expression changes among differentially expressed genes [21].

**Table 2:** Significantly enriched KEGG pathway’s classes for the 109 genes with over-expressed expression and for the 86 genes with under-expressed expression in affected animals versus healthy controls (Count threshold =2, EASE threshold = 0.05)

	**C1 class**	**C2 class**	**C3 class**	**Genes**	**p-value**
**over-expressed**	Cellular Processes	Cell Communication	Focal adhesion	LOC518180, COL4A2, COL1A2, PRKCG, LAMC2, BAD, THBS1, AKT2	0.0005
	Human Diseases	Cancers	Pathways in cancer	LOC518180, DVL2, COL4A2, PRKCG, LAMC2, BCL2L1, BAD, AKT2, DAPK1	0.0021
	Organismal Systems	Nervous System	Neurotrophin signaling pathway	LOC518180, ATF4, SH2B3, BAD, CAMK2A, AKT2	0.0021
	Environmental Information Processing	Signaling Molecules and Interaction	ECM-receptor interaction	COL4A2, SDC1, COL1A2, LAMC2, THBS1	0.0027
	Environmental Information Processing	Signal Transduction	ErbBsignaling pathway	LOC518180, PRKCG, BAD, CAMK2A, AKT2	0.0031
	Human Diseases	Cancers	Chronic myeloid leukemia	LOC518180, BCL2L1, BAD, AKT2	0.020
**under-expressed**	Human Diseases	Neurodegenerative Diseases	Amyotrophic lateral sclerosis (ALS)	DERL1, NEFH, NEFL	0.016

**Figure 1:**
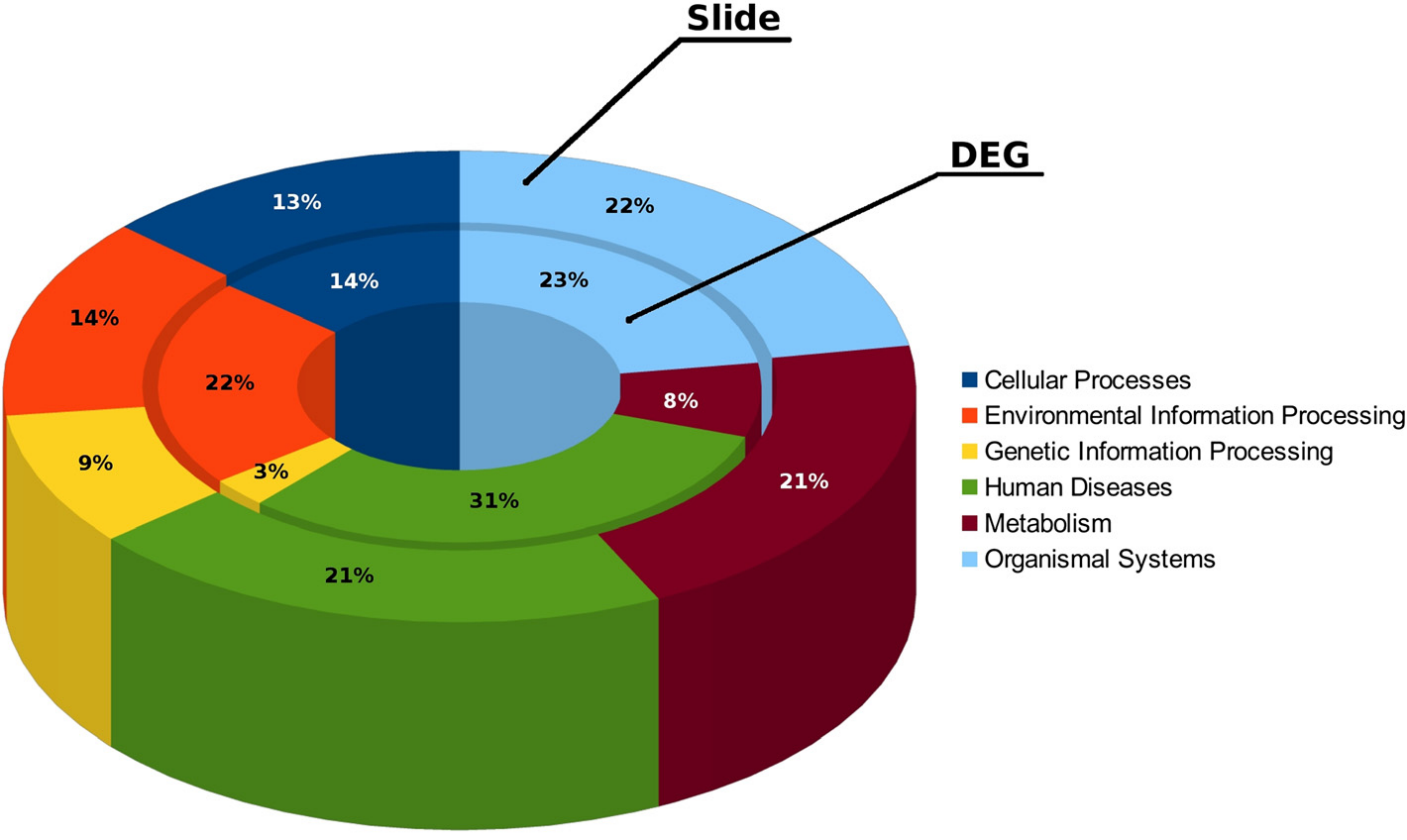
**Gene clusters.** Comparison of clustering between the 195 over- or under-expressed DEGs (|FC| ≥ 2) and the annotated genes on the slide, was assessed using the NCBI DAVID tool. C1 Class KEGG Pathway was selected as the annotation category for clustering.

This clustering tool was first used to check if all the spotted genes from the libraries were uniformly scattered across functional classes, showing a good representation of KEGG Pathways C1 class distribution. The frequency of all genes on the slide and of DEGs (Figure 1) showed that, in KEGG C1 class, the two distributions are significantly independent (P < 0.001).

DAVID was used to analyse genes more than two-fold over- and under-expressed in our samples. Significant functional clustering of affected animals versus healthy controls in KEGG pathways is presented in Table 2. The 109 over-expressed genes belong to Cell Communication, Signaling Molecules and Interaction, Signal Transduction, Cancers and Nervous System C2 classes. Among the 86 genes under-expressed, the Neurodegenerative Diseases KEGG pathway C2 class results the only significant pathway in affected samples.

Two interesting C3 classes, the Amyotrophic Lateral Sclerosis (ALS) and the Neurotrophin signalling pathway are significantly enriched (see *Functional analysis* in Methods). The Amyotrophic Lateral Sclerosis belongs to the C2 class of Neurodegenerative Diseases and includes the under-expressed genes *DERL1*, *NEFH* and *NEFL*. The significant genes belonging to the Neurotrophin signalling pathway (a C2 class of Nervous System) are *AKT2*, *ATF4, BAD*, *CAMK2A* and *SH2B3.* In addition, we observed under expression of *NDUFB2* (FC -2.24) and *TRAPPC4* (FC -2.82) genes which have a role in postsynaptic membrane trafficking and are differentially expressed in Parkinson’s disease pathway [22]. The *BAD*, *NEFH*, *NEFL* and *DERL1* genes are also involved in Huntington’s disease pathways. The Amyotrophic Lateral Sclerosis, the Huntington’s and the Parkinson’s disease pathway pathologies present symptoms partially resembling to BSP’s ones.

### *ATP2A1/SERCA1* and *SLC65* variants causative of BSP in some cattle breeds are not involved in Romagnola BSP

Recently, two genes were associated to other cattle diseases quite similar to spastic paresis: the *ATP2A1*/*SERCA1* gene which encodes for a calcium-transporting ATPase and the *SLC6A5* gene which encodes a presynaptic glycine transporter. In Belgian Blue and in Chianina cattle breeds, the *ATP2A1*/*SERCA1* gene was found to be implicated in the Congenital Muscular Dystonia 1 (CMD1, a disorder of muscle function caused by defects in the Ca^2+^ pump) and in the Congenital Pseudomyotonia [23-26]; while the *SLC6A5* gene was found to be linked to the Congenital Muscular Dystonia 2 (CMD2) [23]. Since in the cDNA microarray we used, these genes were not included, we sequenced regions harbouring the polymorphic sites in the exons 6 and 16 of *ATP2A1*[26] and in the exons 3 and 4 of *SLC6A5*[23]. Both affected and control samples were monomorphic at the examined sites, therefore we can exclude that BSP in Romagnola was caused by the described mutations leading to CMD1 or CMD2 in Chianina and Belgian Blue breeds.

## Discussion

The identification of causative genes associated with a disease is a complex task requiring gathering of genome-wide information. The assessment of the expression of thousands of genes simultaneously may pinpoint the metabolic pathways affected by causative mutation(s) in order to focus on a small set of target genes.

We have tried to assess the metabolic pathways affected by the spastic paresis in Romagnola cattle breed, which presents this disease at quite a high frequency [2], using microarrays. The cDNA microarray we used in this study contains a brain library and, even if carrying an incomplete transcriptome representation, it has proven to be a valuable tool allowing us to add useful and new information to a poorly studied disease. Today, it is possible to prepare custom microarrays [27], while direct sequencing of transcripts by high-throughput sequencing technologies (RNA-Seq) would probably make possible to expand microarray investigation. However by using microarray tool, we analyzed 15 K genes with the aim to acquire knowledge of a genetic disease for which the current literature only provides scant results.

Results on metabolic pathways support an alteration of the Neurodegenerative Diseases and Nervous system C2 classes with the most frequently associated differentially expressed genes in the BSP (*BAD, NEFH, NDUFB2, DERL1, NEFL*). Moreover, the *ATP6V0E1* gene, encoding for a lysosomal H + -transporting ATPase, showed the strongest signal (FC 81.29) on microarrays and together with *NDUFB2*, is involved in intracellular processes and is linked to Oxidative Phosphorylation pathway. *PRKCG* is a member of the protein kinase C (PKC) gene family, whose members phosphorylate a wide variety of protein targets and are known to be involved in diverse cellular signalling pathways. Defects in this protein have been associated with neurodegenerative disorder spinocerebellar ataxia-14 [28].

Four calcium binding proteins involved in Calcium Signalling pathway, *S100A12, S100A11, S100A8* and *STK25,* were weakly over-expressed in the affected animals. In Chianina cattle, a missense mutation in exon 6 (c.491G > A) of the bovine *ATP2A1*/*SERCA1* gene, was implicated in Congenital Pseudomyotonia [24,25], a disease very similar to BSP and paralleled to human Brody disease by Drögemüller and collaborators, [26]. The same gene has been implicated also in Pseudomyotonia in Dutch Improved Red and White cross-breed [29]. We tested this mutation in our diseased animals and we did not find any carrier nevertheless Congenital Pseudomyotonia shares many symptoms with BSP. Therefore, even if BSP does not result associated to the mutations causing Congenital Pseudomyotonia reported in other cattle breeds, our data suggest the involvement of the calcium signalling proteins and two C3 classes, the Neurotrophin signalling pathway and Amyotrophic lateral sclerosis.

Charlier and coll [23] identified a bovine disorder named CMD2, reminiscent of congenital myoclonus in Hereford cattle, and reported a mutation in the glycine transporter GlyT2 (*SLC6A5*) gene already associated to human hyperexplexia [30]. Recently, *SLC6A5* was also associated to hyperexplexia in Irish wolfhounds [31]. BSP shows many similarities with human hyperexplexia (OMIM 149400), an autosomal disease that can be caused by mutations in the genes encoding the alpha-1 subunit of the glycine receptor (*GLRA1*), the presynaptic glycine transporter-2 (*SLC6A5*), the beta-subunit of the glycine receptor (*GLRB*), the postsynaptic glycinergic proteins *GPHN* and *ARHGEF9*[32-39]. Mutations in the latter gene cause hyperexplexia with epilepsy (OMIM 300607). In a previous study, we observed a number of mutations in *GLRA1* and *GLRB* in Romagnola cattle [40], but none resulted significantly involved in the disease.

Our microarray results suggest also a defective glycinergic synaptic transmission in Romagnola BSP. The expression of the Rho GDP dissociation inhibitor (GDI) alpha (*ARHGDIA*) gene, of two neurotransmitter transporters (*SLC41A3* and *SLC25A20),* of the organic anion transporter *SLCO1A2* resulted altered in affected animals. In affected samples, we observed the over-expression of two genes involved in defective glycinergic synaptic transmission, *GRINA* and *SLC6A9*. This latter gene, encoding GlyT1, was recently implicated in defective glycinergic synaptic transmission in Zebrafish [41]. *GFAP*, encoding glial fibrillary acidic protein, linked to expression of the glutamate transporter Glt1 (*SLC1A2*) in Alexander disease, was also over-expressed in affected animals (FC 2.60). Mutations in *NR4A2* (FC 3.06), a nuclear receptor family protein, have been associated with disorders related to dopaminergic dysfunction [42]. Most transporters involved in the drug disposal, characterized by broad substrate specificities and accepting structurally unrelated compounds, include members of SLC family. The involvement of glycinergic proteins seems supported also at phenotypic level: the cholecystokinin (CCK) is involved in the neuroactive ligand-receptor interaction pathway. The release of CCK (ACBP) is linked to GABA and sensitive to clonazepam [43]. These features are found also in human hyperexplexia and are in agreement with the differential expression of some GABA neurotransmitter transporters of the solute carrier family emerging from our analysis.

## Conclusions

BSP is a neuromuscular disorder, known since almost a century, not yet associated with a specific gene. Using microarrays, we identified several genes and pathways significantly linked to the development of BSP in Romagnola cattle breed. We identified genes belonging to two C3 classes, the Neurotrophin signalling pathway and the Amyotrophic Lateral Sclerosis.

Our results suggest also that the development of the disease is linked to a defective glycinergic synaptic transmission and an alteration of calcium signalling proteins. We reviewed the literature on the disease in order to compare published data with the KEGG categories identified by our microarray analysis. This will allow a gene targeted analysis aimed to identify BSP-associated polymorphisms in Romagnola. BSP shares many symptoms with other nervous diseases in this and other species, including humans. The study of the molecular mechanisms underpinning BSP in this and in other cattle breeds, as well as in other livestock species, may help to shed light also on neurodegenerative human diseases.

## Competing interests

The authors declare that they have no competing interests.

## Authors’ contributions

LP: designed the study, carried out microarray experiments and drafted the manuscript. SBu and CEMG: performed microarray probes annotation and KEGG analysis. GP: performed statistical and chromosome position analyses. SBo and SBi: performed PCR and qRT-PCR experiments. AG: performed field survey and provided the samples used in microarray experiments. GC supervised statistical analysis. AV: participated in developing ideas, in supervision and revision of the manuscript. All authors read and approved the final manuscript.

## Supplementary Material

Additional file 1: Table S1 Significantly differentially expressed genes with Fold change |FC| ≥ 2.0. Click here for file

Additional file 2: Table S2 List of primer pairs used for qRT-PCR and PCR. Click here for file
