# Supplementary material for: Microarray gene expression profiling of neural tissues in bovine spastic paresis
Source: BMC Vet Res. 2013 Jun 19;9:122. doi: 10.1186/1746-6148-9-122 (PMC3693873; doi:10.1186/1746-6148-9-122)
Supplement: Additional file 1: Table S1 — Significantly differentially expressed genes with Fold change |FC| ≥ 2.0. [file 1746-6148-9-122-S1.docx]

## Additional file 2 – Table S2. List of primer pairs used for qRT-PCR and PCR

| **Accession**  **no.** | **Forward sequence** | **Reverse sequence** | **Amplicon size (bp)** |
| --- | --- | --- | --- |
| *Beta-actin* (BC142413.1) | GTCCACCTTCCAGCAGATGT | AAAGCCATGCCAATCTCATC | 178 |
| *ATP6V01* (CO874887) | TCTGGTACCTCAAGTATCATTGGCC | TCAAGGAGGTGGTCTGGGTACTG | 122 |
| *S100A12* (CK778716) | CGTCTTTATCGGCATCCAGGTCTTG | GCATTTCGACACCCTCAACAAGCG | 129 |
| *BCL2L1* (CO895351) | GGAGATCGAACAGCCTGATGC | ACTGCACCTTTACTTTCACACAGAC | 172 |
| *ATP2A1* exon 6  (ENSBTAG00000006541) | TGGGGTTTTCTCCCTTCTCT | AGGCTGTGAGCAAAGCTGA | 292 |
| *ATP2A1* exon 16  (ENSBTAG00000006541) | CTCAGCTTGCGGTCTGCTCTGAC | TGGTGCCCACTCGCACGTAGT | 439 |
| *SLC6A5* exon 3  (ENSBTAG00000014300) | CAAAAGCTTCCTGGAGAAGG | GGGTTCTCCCAATCACACAA | 337 |
| *SLC6A5* exon 4  (ENSBTAG00000014300) | CTGTAAGAGGGAGCCAGCAC | CATCTCTGTACCCCCAGCAT | 401 |
