# Supplementary material for: Microarray gene expression profiling of neural tissues in bovine spastic paresis
Source: BMC Vet Res. 2013 Jun 19;9:122. doi: 10.1186/1746-6148-9-122 (PMC3693873; doi:10.1186/1746-6148-9-122)
Supplement: Additional file 2: Table S2 — List of primer pairs used for qRT-PCR and PCR. [file 1746-6148-9-122-S2.docx]

## Additional file 1 – Table S1. Significantly differentially expressed genes with Fold change ≥|2.0|.

| **gb_acc** | **FC** | **p.value** | **gene** |
| --- | --- | --- | --- |
| CO874887 | 81.293 | 0.001 | *ATP6V0E1* |
| CK778716 | 23.713 | 0.005 | *S100A12* |
| BE683422 | 21.653 | 0.000 | *CATHL1* |
| CO878659 | 5.9 | 0.000 | *HIST2H2AC* |
| CO885408 | 4.758 | 0.002 | *LOC100140532* |
| CO887972 | 4.116 | 0.000 | *HCN2* |
| BM256666 | 3.684 | 0.010 | *S100A8* |
| CO892153 | 3.674 | 0.024 | *AGBL5* |
| CO887732 | 3.526 | 0.011 | *LOC789538* |
| CO874773 | 3.274 | 0.023 | *OLFM1* |
| CO875481 | 3.233 | 0.006 | *BHLHE22* |
| CO883808 | 3.2 | 0.007 | *ZNF282* |
| CO872063 | 3.169 | 0.047 | *PLEKHA4* |
| CO895605 | 3.163 | 0.005 | *CAMK2A* |
| CO896957 | 3.146 | 0.012 | *STK24* |
| BM256725 | 3.13 | 0.005 | *GZMH* |
| CO895275 | 3.063 | 0.004 | *NR4A2* |
| CO890384 | 2.981 | 0.000 | *COL1A2* |
| CO879915 | 2.929 | 0.014 | *PNMA2* |
| CO892662 | 2.92 | 0.007 | *DIDo1* |
| CO893225 | 2.904 | 0.000 | *SETD1A* |
| CO886432 | 2.889 | 0.037 | *EIF3K* |
| CO894362 | 2.884 | 0.005 | *FBXW5* |
| CO886101 | 2.879 | 0.034 | *SCRN1* |
| CO885555 | 2.851 | 0.035 | *MYH14* |
| CO893333 | 2.851 | 0.008 | *NCLN* |
| CO889542 | 2.779 | 0.018 | *PIP5K1C* |
| CO886887 | 2.767 | 0.008 | *STK25* |
| CO893063 | 2.755 | 0.024 | *PTPLAD1* |
| CO885154 | 2.755 | 0.023 | *ES1* |
| CO882775 | 2.725 | 0.007 | *CNTNAP1* |
| CO895865 | 2.721 | 0.030 | *MIB2* |
| CO883580 | 2.7 | 0.001 | *LOC100059767* |
| AJ820983 | 2.681 | 0.001 | *CUTC* |
| CO893738 | 2.628 | 0.012 | *TIMP1* |
| CO873484 | 2.624 | 0.040 | *LOC520718* |
| CO893094 | 2.621 | 0.011 | *LOC518180* |
| CO890979 | 2.606 | 0.006 | *AP2A2* |
| CO892268 | 2.606 | 0.035 | *RN18S1* |
| CO883102 | 2.599 | 0.005 | */* |
| CO873005 | 2.598 | 0.025 | *GFAP* |
| CO878976 | 2.589 | 0.041 | *VOPP1* |
| CO879966 | 2.583 | 0.009 | *IGL@* |
| CO881956 | 2.574 | 0.017 | *MAEA* |
| CO895781 | 2.566 | 0.007 | *MAEA* |
| CO894846 | 2.559 | 0.008 | *LMTK3* |
| CO897017 | 2.555 | 0.015 | */* |
| CO879871 | 2.53 | 0.020 | *RAB3IL1* |
| CO878864 | 2.523 | 0.001 | *SCRT1* |
| CO889707 | 2.52 | 0.018 | *LTBP4* |
| BM482417 | 2.52 | 0.002 | *THBS1* |
| CO887201 | 2.508 | 0.002 | *H2AFJ* |
| S0000049F10 | 2.488 | 0.001 | */* |
| CO884552 | 2.472 | 0.001 | *TMEM63C* |
| CO891794 | 2.462 | 0.007 | *SGSM3* |
| CO891039 | 2.454 | 0.020 | *MIB2* |
| CO876026 | 2.448 | 0.006 | *DPF1* |
| CO890166 | 2.445 | 0.021 | *C17H12orf43* |
| CO888085 | 2.441 | 0.018 | *SIGMAR1* |
| CK846212 | 2.426 | 0.001 | *BAD* |
| CO880991 | 2.393 | 0.045 | *Transcribedlocus* |
| CO876778 | 2.361 | 0.007 | *SLC41A3* |
| CO883193 | 2.352 | 0.003 | *Transcribedlocus* |
| CO881022 | 2.349 | 0.007 | *LOC786966* |
| CO886116 | 2.346 | 0.041 | *LOC515452* |
| CO895870 | 2.344 | 0.014 | *MRPS34* |
| AJ813615 | 2.344 | 0.001 | *SPATA2* |
| CO891310 | 2.342 | 0.001 | *LIPE* |
| CO874947 | 2.34 | 0.020 | *REXO1* |
| CO895351 | 2.337 | 0.038 | *BCL2L1* |
| CO894830 | 2.332 | 0.028 | *MECR* |
| CO893780 | 2.322 | 0.022 | *SNAP29* |
| AJ818369 | 2.316 | 0.029 | *FARS2* |
| CO892142 | 2.312 | 0.001 | *LOC783074* |
| CO890666 | 2.299 | 0.010 | *LOC510613* |
| MARC_1BOV_79 | 2.287 | 0.001 | */* |
| AJ814701 | 2.287 | 0.035 | *LOC100337053* |
| CO872313 | 2.282 | 0.042 | *AKT2* |
| CO880968 | 2.282 | 0.031 | *PPP2R4* |
| CO883531 | 2.279 | 0.023 | *SDC1* |
| CO889680 | 2.277 | 0.031 | *GUCY1A2* |
| CO893724 | 2.271 | 0.003 | *NRXN2* |
| CO881849 | 2.27 | 0.027 | *MIB2* |
| CO883820 | 2.26 | 0.027 | *CHPF* |
| CO873098 | 2.257 | 0.001 | *Transcribedlocus* |
| CO880475 | 2.255 | 0.007 | *DYM* |
| CO895053 | 2.245 | 0.007 | *ROBLD3* |
| CO893624 | 2.232 | 0.004 | *COL4A2* |
| CO889220 | 2.227 | 0.047 | *LAMC2* |
| CO886689 | 2.221 | 0.018 | *GAS6* |
| AJ814728 | 2.22 | 0.016 | *BLNK* |
| AJ819849 | 2.217 | 0.025 | */* |
| CO887402 | 2.212 | 0.020 | *Transcribedlocus* |
| CK772673 | 2.207 | 0.015 | *CCR9* |
| CO894316 | 2.195 | 0.003 | *Transcribedlocus* |
| CO889797 | 2.185 | 0.048 | *ARHGDIA* |
| S0000049A11 | 2.182 | 0.004 | */* |
| CO885416 | 2.179 | 0.002 | *pou2f1* |
| CO880556 | 2.178 | 0.006 | *PNPLA2* |
| CO896746 | 2.178 | 0.010 | *SCAMP2* |
| CO892154 | 2.175 | 0.001 | *DNAJC14* |
| CO875636 | 2.168 | 0.009 | *TRABD* |
| BE751386 | 2.157 | 0.032 | *ADM* |
| CO883460 | 2.155 | 0.016 | *CHMP4B* |
| CO887762 | 2.153 | 0.046 | */* |
| CO892655 | 2.148 | 0.038 | *ATF4* |
| CO888985 | 2.145 | 0.022 | *LOC100125939* |
| AJ817793 | 2.141 | 0.045 | *PIK3C2B* |
| CO872039 | 2.141 | 0.028 | *CRIP2* |
| CO881160 | 2.135 | 0.003 | *TMEM170B* |
| CO885648 | 2.115 | 0.027 | *PKIG* |
| CO877660 | 2.106 | 0.049 | *PRKCG* |
| CO891528 | 2.104 | 0.006 | *KCNC4* |
| CK772767 | 2.102 | 0.005 | *DAPK1* |
| CO879535 | 2.101 | 0.046 | *LAPTM4B* |
| CO885145 | 2.1 | 0.005 | */* |
| CO894454 | 2.097 | 0.011 | *HSF1* |
| CO896210 | 2.093 | 0.029 | *GOLPH3L* |
| BM256607 | 2.093 | 0.033 | *HOXA9* |
| CO872349 | 2.087 | 0.022 | *TXLNA* |
| CO895775 | 2.085 | 0.007 | *EPHB6* |
| CO890080 | 2.084 | 0.004 | *BCL2L12* |
| CO889317 | 2.069 | 0.001 | *BAIAP2* |
| MARC_1BOV_85 | 2.05 | 0.030 | */* |
| CO895200 | 2.048 | 0.011 | *S100A11* |
| CO877313 | 2.046 | 0.043 | *OSBPL5* |
| CO894700 | 2.046 | 0.038 | *SH2B3* |
| CO885211 | 2.044 | 0.025 | *EPHA7* |
| CO896917 | 2.043 | 0.009 | *DVL2* |
| CO871920 | 2.041 | 0.045 | *LA-DRB* |
| CO888924 | 2.039 | 0.003 | *LOC100849810* |
| CO896381 | 2.037 | 0.001 | *SHANK3* |
| CO875841 | 2.029 | 0.039 | *LOC515954* |
| CO894504 | 2.026 | 0.040 | *NFATC1* |
| CO896297 | 2.015 | 0.006 | *SYT5* |
| CO882466 | 2.013 | 0.012 | *BRSK1* |
| CO892325 | 2.005 | 0.018 | *PACSIN2* |
| CO880473 | 2.001 | 0.031 | *STK11* |
| CO877544 | 2 | 0.006 | *FBXO2* |
| CO891964 | -2.001 | 0.005 | *RORC* |
| CO893261 | -2.006 | 0.047 | *TFCP2* |
| CO893634 | -2.007 | 0.024 | *APBB2* |
| CO880398 | -2.007 | 0.041 | *GCLC* |
| CO879783 | -2.007 | 0.002 | *PRPF39* |
| CO893594 | -2.008 | 0.018 | *LOC100851966* |
| CO881443 | -2.012 | 0.012 | *NPAL2* |
| CO894844 | -2.018 | 0.009 | */* |
| AJ814635 | -2.019 | 0.006 | *SSB* |
| CO874418 | -2.02 | 0.000 | *ANXA5* |
| AJ820458 | -2.023 | 0.006 | *PC* |
| CO895551 | -2.028 | 0.007 | *RBM5* |
| AJ816085 | -2.029 | 0.013 | *SYN1* |
| CO889786 | -2.033 | 0.037 | *P2X* |
| AJ816196 | -2.036 | 0.018 | *SH2D3C* |
| CO889923 | -2.036 | 0.005 | */* |
| CO890430 | -2.042 | 0.008 | *HNRNPA2B1* |
| AJ813445 | -2.042 | 0.032 | *SETMAR* |
| CO890759 | -2.045 | 0.001 | *LOC511936* |
| CO875698 | -2.047 | 0.004 | *DOCK6* |
| AJ813526 | -2.047 | 0.012 | *BCLAF1* |
| CO872670 | -2.05 | 0.001 | *IMMP1L* |
| CO874541 | -2.051 | 0.001 | *MT3* |
| AJ820838 | -2.052 | 0.005 | *ARID5B* |
| CO883423 | -2.053 | 0.013 | *LOC100847863* |
| CO892613 | -2.053 | 0.012 | *DNAJB2* |
| AJ815922 | -2.054 | 0.008 | *NUDT15-like* |
| CO887676 | -2.058 | 0.011 | *LOC532875* |
| CO878775 | -2.061 | 0.042 | *SHROOM2* |
| CO890225 | -2.062 | 0.002 | *LOC100850008* |
| CO875149 | -2.072 | 0.048 | *NEFH* |
| CO887092 | -2.074 | 0.022 | *LOC100847863* |
| CO878548 | -2.094 | 0.004 | *DGAT2* |
| CO885678 | -2.094 | 0.002 | *NSA2* |
| CO883416 | -2.095 | 0.006 | *NSA2* |
| CO893909 | -2.104 | 0.001 | *EFCAB6* |
| AJ813429 | -2.107 | 0.024 | */* |
| CO883833 | -2.109 | 0.010 | *GCLC* |
| AJ814525 | -2.111 | 0.036 | *KIF13B* |
| CO887392 | -2.113 | 0.003 | *SERPINE2* |
| CO887735 | -2.113 | 0.005 | *LOC100851748* |
| CO896071 | -2.116 | 0.000 | */* |
| CO895348 | -2.123 | 0.035 | *GMEB1* |
| CO891283 | -2.127 | 0.006 | *Transcribedlocus* |
| AJ816725 | -2.127 | 0.027 | *DENND5B* |
| AJ818732 | -2.128 | 0.002 | */* |
| CO882194 | -2.131 | 0.037 | */* |
| CO880976 | -2.133 | 0.014 | */* |
| AJ813936 | -2.144 | 0.006 | *HNMT* |
| CO886377 | -2.149 | 0.003 | *ALDH1L2* |
| CO889086 | -2.152 | 0.001 | *REEP5* |
| AJ813254 | -2.153 | 0.001 | */* |
| CO886663 | -2.156 | 0.015 | *KIAA0681* |
| CO886226 | -2.156 | 0.027 | *TNFRSF19* |
| AJ813513 | -2.16 | 0.016 | *ALG6* |
| AJ819520 | -2.161 | 0.004 | *HIF1A* |
| CO894697 | -2.166 | 0.026 | *CAMLG* |
| CO879578 | -2.176 | 0.002 | */* |
| AJ821024 | -2.182 | 0.025 | */* |
| AJ818590 | -2.183 | 0.001 | */* |
| AJ820591 | -2.185 | 0.002 | *CCDC69* |
| AJ813649 | -2.185 | 0.003 | *ZNF354A* |
| CO886052 | -2.187 | 0.000 | */* |
| CO896747 | -2.19 | 0.013 | *TCRG1* |
| CO885520 | -2.191 | 0.002 | *SLC25A20* |
| CO878082 | -2.198 | 0.010 | *USE1* |
| AJ813657 | -2.201 | 0.001 | *PTPN9* |
| CO882130 | -2.203 | 0.000 | *ATP1B4* |
| CO873194 | -2.207 | 0.001 | *LUC7L3* |
| CO883325 | -2.209 | 0.000 | *YKT6* |
| AJ814464 | -2.213 | 0.008 | */* |
| CO889410 | -2.213 | 0.001 | *Transcribedlocus* |
| AJ813129 | -2.225 | 0.011 | *BMP-2* |
| AJ813796 | -2.23 | 0.019 | */* |
| AJ817688 | -2.236 | 0.014 | */* |
| AJ815995 | -2.239 | 0.003 | *APIP* |
| CO887095 | -2.24 | 0.001 | *SFRS18* |
| CO886483 | -2.241 | 0.018 | *NDUFB2* |
| CO885166 | -2.245 | 0.000 | */* |
| CO891504 | -2.247 | 0.047 | *LOC784171* |
| CO885046 | -2.257 | 0.010 | */* |
| AJ817012 | -2.263 | 0.008 | */* |
| CO894678 | -2.271 | 0.018 | *CHRNB1* |
| CO881831 | -2.274 | 0.017 | *DERL1* |
| CO877816 | -2.279 | 0.003 | *SMC5* |
| CO883821 | -2.281 | 0.001 | *MOG* |
| CO892547 | -2.284 | 0.026 | */* |
| CO895952 | -2.304 | 0.043 | *SFRS5* |
| CO889544 | -2.31 | 0.007 | *FGD4* |
| CO880538 | -2.311 | 0.001 | *MUC16* |
| AJ819179 | -2.324 | 0.027 | *MUC16* |
| CO892194 | -2.358 | 0.000 | *GHRL* |
| CO894894 | -2.374 | 0.008 | *ZNF180* |
| AJ817660 | -2.414 | 0.003 | *GSTM3* |
| CO895224 | -2.422 | 0.007 | *PDCD6* |
| CO891303 | -2.423 | 0.003 | *PEX26* |
| CO878830 | -2.423 | 0.003 | *ZNF879* |
| AJ816528 | -2.455 | 0.047 | *DOCK4* |
| CO888593 | -2.462 | 0.003 | *LOC535121* |
| CO887361 | -2.468 | 0.007 | *FAM33A* |
| CO892414 | -2.489 | 0.003 | *CREB5* |
| CO895631 | -2.507 | 0.026 | *TNS4* |
| CO884554 | -2.508 | 0.000 | *CATSPERG* |
| CO893097 | -2.517 | 0.003 | *SEC24C* |
| AJ820719 | -2.535 | 0.003 | *RUNX1* |
| CO879805 | -2.547 | 0.001 | *TTRAP* |
| CO890969 | -2.555 | 0.013 | *TCRG1* |
| AJ818175 | -2.559 | 0.017 | *ANGPTL2* |
| CO892472 | -2.566 | 0.022 | *BMPR1B* |
| CO893053 | -2.575 | 0.019 | *NRP* |
| CO877207 | -2.587 | 0.005 | *LOC787355* |
| CO893245 | -2.593 | 0.001 | *LOC509490* |
| CO881481 | -2.607 | 0.013 | *COX* |
| AJ816376 | -2.618 | 0.004 | *GPHN* |
| AJ821017 | -2.633 | 0.006 | */* |
| CO894865 | -2.684 | 0.015 | *CSNK2A2* |
| AJ821004 | -2.700 | 0.000 | *RBM6* |
| CO882263 | -2.703 | 0.001 | *ZFC3H1* |
| CO884789 | -2.724 | 0.041 | *NEFL* |
| CO887281 | -2.76 | 0.007 | *ND6* |
| CO878157 | -2.764 | 0.006 | *LOC100138404* |
| CO886908 | -2.778 | 0.033 | *ARC* |
| CO884142 | -2.823 | 0.011 | *TRAPPC4* |
| CO892444 | -2.824 | 0.001 | *PRNP* |
| CO894801 | -2.826 | 0.028 | *CREBZF* |
| CO890269 | -2.918 | 0.023 | *SMARCA5* |
| CO890292 | -3.302 | 0.001 | *ESD* |
| CO897037 | -3.425 | 0.001 | *PRL* |
| CO888880 | -4.373 | 0.017 | *ESD* |
